# Radial spin echo small-angle neutron scattering method: concept and performance

**DOI:** 10.1107/S1600576722007245

**Published:** 2022-08-24

**Authors:** Elisabeth Kadletz, Wim G. Bouwman, Catherine Pappas

**Affiliations:** a Delft University of Technology, Faculty of Applied Sciences, The Netherlands; Tohoku University, Japan

**Keywords:** small-angle neutron scattering, polarized neutrons, spin echo small-angle neutron scattering, Larmor labelling

## Abstract

A novel spin echo small-angle neutron scattering (SESANS) concept based on a rotationally symmetric magnetic field geometry is introduced. The proposed method is similar to the conventional linear SESANS technique but uses longitudinal precession fields and field gradients in a radial direction, as typically found in neutron spin echo (NSE) spectrometers. Radial SESANS could thus be implemented as an add-on to NSE setups. The neutron trajectory through the instrument is encoded with the help of radial gradients generated by radial shifters, which are coils placed in the beam area similar to Fresnel coils. The present work introduces the setup of the instrument and explores its performance and the relationship between the encoded momentum transfer and the precession angle. The results indicate that radial SESANS is only sensitive to scattering along the radial direction and thus measures the projected correlation function along this direction as a function of the spin echo length, defined similarly to linear SESANS. For an evaluation of the performance of the setup, the case of scattering from solid spheres is considered and the results calculated for the radial and linear SESANS cases are compared. Also discussed is the implementation of the radial magnetic field geometry in spin echo modulated small-angle neutron scattering.

## Abstract 2

The concept of a radially symmetric spin echo small-angle neutron scattering (SESANS) setup is introduced and analyzed.

## 1. Introduction

In neutron scattering, the energy and momentum of the neutron beam are usually measured both before and after scattering in order to determine the energy or momentum transfer at the sample. Achieving a high accuracy of these measurement parameters requires high collimation and monochromatization which, in turn, leads to a dramatic loss in beam intensity and thus limits the practically achievable resolution. This limitation can be overcome by neutron spin echo techniques. These use the Larmor precession of polarized neutrons in a magnetic field to measure directly the transfer of energy ω or momentum **Q** [*Q* = (4π/λ)sin(θ/2), where θ is the scattering angle and λ is the wavelength of the incident radiation] at the sample without the need to determine these quantities independently for the incoming and scattered neutron beams. In this way, the accuracy with which ω or **Q** are determined becomes independent of beam characteristics, and very high resolutions can be reached without the prohibiting low-intensity penalty of other neutron scattering methods.

### 1.1. Basics of neutron spin echo spectroscopy

**Figure 1:**
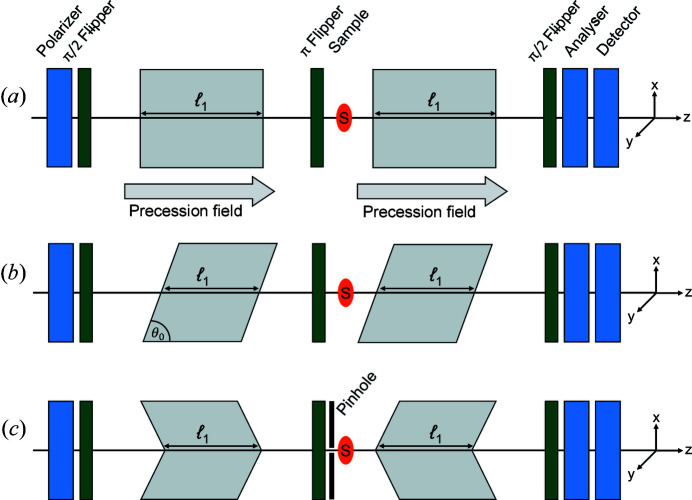
Cross-sectional views of the discussed setups showing the shape of the effective precession regions. (*a*) An NSE spectrometer with homo­geneous magnetic field integrals shown as flat precession field area boundaries. (*b*) Linear SESANS with shifters generating a gradient in the magnetic field integral along the *x* direction, as illustrated by the inclined front and end faces of the precession field areas. This setup works with longitudinal or transverse precession fields. (*c*) A SESANS instrument with radial shifters, which generate a radial gradient of the magnetic field integral gradient with rotational symmetry about the optical axis of the instrument. This is illustrated by the conical front and end faces of the precession field area boundaries.

Neutron spin echo (NSE) spectroscopy was first introduced by Mezei (1972 ▸) to measure the energy transfer in inelastic scattering. In an NSE spectrometer, a polarized neutron beam traverses two equal magnetic precession regions in which it experiences Larmor precession in opposite directions. A simplified NSE setup is shown in Fig. 1 ▸(*a*). The π/2 spin flipper at the beginning of the first precession region turns the polarization of the neutron beam **P** perpendicular to the magnetic field **B**. This induces Larmor precession with the characteristic Larmor frequency ω_L_ = γ*B*, where γ = 1.832 × 10^8^ s^−1^ T^−1^ is the neutron gyromagnetic ratio. For a neutron beam propagating with a speed *v* over a path of length ℓ, where a constant magnetic field of strength *B* is applied, the accumulated precession phase φ is given by (Mezei, 1980*c* ▸)

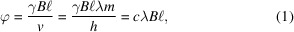

with λ the wavelength, *m* the neutron mass, *h* the Planck constant and *c* = 4.636 × 10^14^ T^−1^ m^−2^. The precession angle φ is thus proportional to both the neutron beam wavelength and the magnetic field integral (along the neutron trajectory), which in our simplified case (homogeneous magnetic field, highly collimated, *i.e.* non-divergent, neutron beam) reduces to the product of *B* and ℓ.

A π flipper at the symmetry point of the setup, which is close to the sample position, reverses the precession direction in the second field area. Consequently, if the two precession regions are identical, the precession angles before and after the sample cancel each other. This leads to the spin echo condition Δφ = φ_incoming_ − φ_scattered_ = 0 at the second π/2 flipper, which is positioned at the end of the second precession region in front of the analyser and the detector. This flipper stops the precession and turns **P** in the direction of the magnetic field and thus of the analyser, so that ideally the maximum intensity is recovered at the detector (Mezei, 1980*c* ▸; Mezei *et al.*, 2003 ▸).

Inelastic scattering from the sample breaks the symmetry of the setup as the energy transfer ω modifies the speed, and thus the wavelength, of the scattered neutron beam. As a result, the precession phases accumulated before and after the sample are no longer equal to each other, leading to a non-zero total precession phase Δφ and a reduction in the detected intensity. In the limit, where the energy transfer at the sample is much smaller than the energy of the incoming neutron beam, Δφ is directly proportional to ω, 

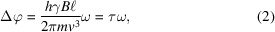

where the proportionality factor τ is the Fourier time (Mezei, 1980*c* ▸). The component of the beam polarization **P** transmitted by the analyser is proportional to 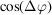 . Thus, the net precession angle Δφ is reflected in the normalized spin echo signal determined by integrating over all possible energy transfers, which is equal to the intermediate scattering function (Mezei, 1980*c* ▸): 

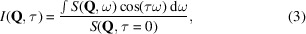

where *S*(**Q**, ω) 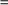 d^2^Σ/d**Q**dω, with Σ the total scattering cross section of the sample, and 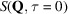 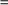 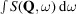 is the static structure factor. In other words, NSE measures the real (cosine) part of the ω Fourier transform of *S*(**Q**, ω) and thus the τ dependence of the correlation function (Gähler *et al.*, 1996 ▸; Mezei *et al.*, 2003 ▸).

### 1.2. Linear SESANS

The same principles of Larmor precession and neutron spin echo can be applied in experiments with elastic scattering [*i.e.* for ω = 0, in which case *S*(**Q**, ω) reduces to *S*(**Q**) = dΣ(**Q**)/d**Q**] to measure the scattering angle and thus the momentum transfer. This labelling of a neutron beam trajectory by the Larmor precession phase (Pynn, 1978 ▸, 1980 ▸) has been applied for small-angle neutron scattering (SANS), neutron reflectivity and neutron diffraction (Rekveldt *et al.*, 2003 ▸), leading to an increase in resolution by several orders of magnitude compared with conventional neutron scattering methods.

Encoding the neutron trajectory using the Larmor precession is possible by introducing specially shaped precession regions with inclined front and end faces, as shown in Fig. 1 ▸(*b*). This is achieved by adding linear field gradients that span the whole beam cross section. Analogously to NSE, the neutron polarization undergoes Larmor precession in opposite directions in two precession regions before and after the sample. Furthermore, the magnetic field configuration and arrangement of the gradients is such that the spin echo condition is satisfied in the absence of scattering.

The accumulated total precession phase averaged over the neutron beam now also depends on the linear gradient, which leads to different phases for different trajectories through the instrument. The vertically inclined field boundaries lead to a linear relation between φ and the vertical transmission angle θ_*x*_ of the neutron beam. If there is scattering at the sample, the transmission angle changes and, in the small-angle scattering approximation, the difference in transmission angles Δθ = θ_incoming_ − θ_scattered_ directly yields the momentum transfer for elastic scattering. Therefore, the net precession angle encodes the momentum transfer along the linear gradient *Q*_*x*_ (Rekveldt *et al.*, 2003 ▸, 2005 ▸; Bouwman *et al.*, 2000 ▸), 

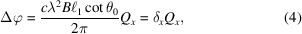

where θ_0_ is the inclination angle of the field boundaries. The quantity δ_*x*_ is the spin echo length, which is proportional to λ^2^ and the magnetic field integral.

In the following we focus on isotropic elastic scattering and on the spin echo small-angle neutron scattering (SESANS) (Rekveldt, 1996 ▸) realization of this method. We designate the geometry of Fig. 1 ▸(*b*) as linear SESANS. As the measurements are performed in the direct beam it is convenient to consider the average spin echo polarization, 

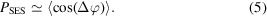

In the case of scattering from a sample with total scattering cross section Σ and thickness *t*, *P*_SES_ is related to the SESANS correlation function *G*(δ_*x*_),

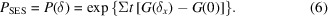

In the small-angle approximation, the total cross section is given by 

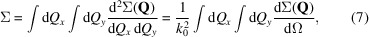

with *k*_0_ = 2π/λ and Ω the probed solid angle. This description includes the fraction of the neutron beam that does not scatter, as well as all scattering including multiple scattering (Rekveldt *et al.*, 2003 ▸).

The normalized SESANS correlation function *G*(δ_*x*_) is given by 

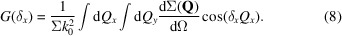




**Figure 2:**
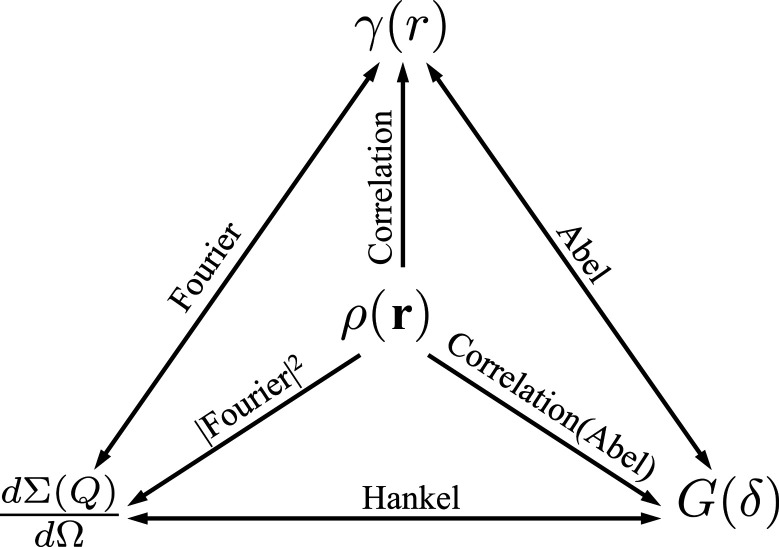
A transform triangle illustrating the relations between the scattering function dΣ(**Q**)/dΩ, density autocorrelation function γ(*r*) and projected correlation function *G*(δ) for an isotropic density distribution ρ(*r*). Adapted from Andersson *et al.* (2008 ▸).

Equations (6) and (8) show that, similarly to NSE, SESANS measures a function which involves the Fourier transform of the scattering cross section dΣ(**Q**)/dΩ. However, the integration in this case is with respect to the momentum transfer, which is a two-dimensional vector in the detector plane. Consequently, for isotropic density distributions ρ(*r*) the measured *G*(δ_*x*_) and dΣ(**Q**)/dΩ are connected by a Hankel transform (Krouglov, de Schepper *et al.*, 2003 ▸; Andersson *et al.*, 2008 ▸; Kohlbrecher & Studer, 2017 ▸), as illustrated in Fig. 2 ▸. *G*(δ_*x*_) is the projection of the normalized density autocorrelation function γ(*r*) along the neutron path (Andersson *et al.*, 2008 ▸; Krouglov, de Schepper *et al.*, 2003 ▸; Kohlbrecher & Studer, 2017 ▸), 

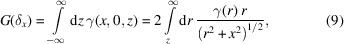

with *z* the coordinate parallel to the beam propagation, *x* the coordinate along the magnetic gradients [as shown in Fig. 1 ▸(*b*)] and *r* = (*x* ^2^ + *z* ^2^)^1/2^.

The described SESANS setup is not sensitive in the *y* direction and only measures correlations along *x*, the direction of the magnetic field integral gradient. In the second step in equation (9), a coordinate transformation from *x*, *z* to *x*, *r* is performed, and the resulting formula shows that *G*(δ_*x*_) is an Abel transform of γ(*r*) (Krouglov, de Schepper *et al.*, 2003 ▸).

The first dedicated SESANS instrument was built at TU Delft (Rekveldt, 1996 ▸) and uses transverse precession fields, *i.e.* perpendicular to the optical axis *z*, which are generated by permanent magnets. SESANS finds a broad range of applications such as in colloid science (Krouglov, Bouwman *et al.*, 2003 ▸, 2005 ▸; Li *et al.*, 2011 ▸; Washington *et al.*, 2014 ▸; van Gruijthuijsen *et al.*, 2014 ▸, 2018 ▸) or food science (Bouwman, 2021 ▸). Most SESANS instruments access spin echo lengths spanning three orders of magnitude from 10 nm up to 20 µm. The maximum length that can be probed is two orders of magnitude larger than that in conventional SANS. Besides the increased resolution, a significant advantage is the simple consideration of multiple scattering (Rekveldt *et al.*, 2003 ▸). An interesting feature, especially for the communication of the data to non-scattering experts, is the real-space character of the direct measurements (Bouwman *et al.*, 2008 ▸).

A more recent variation in the technique is spin echo modulation small-angle neutron scattering (SEMSANS) (Bouwman *et al.*, 2009 ▸, 2011 ▸; Sales *et al.*, 2015 ▸; Strobl *et al.*, 2015 ▸). A SEMSANS setup consists of only two precession devices with different gradients that create a spatial modulation of intensity at a detector. All spin manipulations can be performed before the sample, which gives more freedom to work with magnetic samples (Li *et al.*, 2021 ▸) or sample environments. SEMSANS can also be combined with normal SANS to probe a much longer range of length scales (Schmitt *et al.*, 2020 ▸).

### 1.3. Introducing radial SESANS

When it was first proposed to use the neutron spin echo principle for structural investigations, it was considered that the method would deliver the Fourier transform of dΣ(**Q**)/dΩ (Pynn, 1978 ▸, 1980 ▸; Gähler *et al.*, 1996 ▸) closely following the mathematical concepts of NSE. This would imply that linear SESANS would measure the three-dimensional autocorrelation function directly (Gähler *et al.*, 1998 ▸). However, it turns out that only one direction, the one along the magnetic field gradient, is probed. This leads to a two-dimensional projection of the autocorrelation function and to the Hankel transform between *G*(δ_*x*_) and dΣ(**Q**)/dΩ discussed above.

A method for direct measurement of the density autocorrelation function would also be of interest because it would facilitate the comparison between SANS and SESANS data sets. As a key feature, such a SESANS method should be able to measure *Q*, the modulus of the total momentum transfer, not just the one component along a certain direction. For this purpose one might consider introducing radially symmetric magnetic field precession regions, which would allow for measuring correlations independent of a specific direction. This concept is discussed in the following and is referred to as radial SESANS. In order to respect the radial symmetry of the setup, we consider longitudinal precession fields with radially symmetric gradients as shown schematically in Fig. 1 ▸(*c*). This radial symmetry is also reflected in the precession angle which, as discussed in the following section, depends on the radial distance of the neutron path from the optical axis.

**Figure 3:**
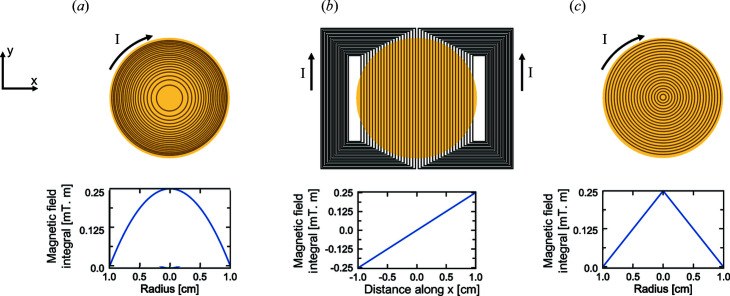
Comparison between (*a*) a Fresnel correction coil, (*b*) a linear shifter and (*c*) a radial shifter. On the top row are illustrations of the coils with the active beam area shown in yellow. On the bottom row, the magnetic line integrals are plotted over the cross-sectional area. A current of *I* = 10 A and a wire density in the coils of *n*_W_ = 2 mm^−1^ were assumed.

Such a setup was first discussed by Zhao (2001 ▸) with the aim of developing a SESANS method where the density distribution function would be the simple Fourier transform of dΣ(**Q**)/dΩ, similar to NSE spectroscopy. The radial SESANS setup discussed by Zhao uses longitudinal magnetic fields created by solenoids similar to those of NSE spectrometers with in-beam gradient coils. Therefore, it can be considered as a possible add-on for existing NSE spectrometers. In NSE, Fresnel coils (Mezei, 1980*a* ▸; Monkenbusch, 1990 ▸), shown schematically in Fig. 3 ▸(*a*), are used to correct the magnetic field integral inhomogeneities inherent to divergent beams with large cross sections. Similarly, SESANS could use shifter coils, linear or radial [see Figs. 3 ▸(*b*) and 3 ▸(*c*), respectively], not to correct for magnetic field inhomogeneities but to encode the neutron trajectory by adding a shift to the precession angle depending on a specific neutron trajectory. Schematic drawings of the resulting configurations are shown in Figs. 1 ▸(*b*) and 1 ▸(*c*) for linear and radial SESANS, respectively.

In this paper we investigate the realization conditions and the performance of a radial SESANS setup for small-angle neutron scattering. Our calculations are based on parameters which are compatible with state-of-the-art NSE spectrometers, such as the IN15 instrument at the Institute Laue–Langevin (Schleger *et al.*, 1999 ▸). The focus is on the measured momentum transfer and the obtained correlation function.

Our findings show that in radial SESANS the labelling of the trajectories, and thus of the momentum transfer, is along the radial directions. However, it is not possible to obtain the total momentum transfer from the measured signal as radial SESANS is also sensitive to only one component of the momentum transfer vector. Thus, in this case we also obtain a projected correlation function along the radius, which is similar to linear SESANS. This implies that, regardless of the symmetry, radial or linear, SESANS probes correlation along only one direction, which is the direction of the field gradient. We discuss the implications of this result for other scattering geometries.

## 2. Radial SESANS

As discussed above, when considering the implementation of SESANS on NSE spectrometers, where the magnetic fields are generated by solenoids and their direction is along the optical axis of the instrument (*i.e.* longitudinal), there are two possibilities to trace the neutron trajectory through the device, either with linear shifters or with radial shifters, as shown in Figs. 3 ▸(*b*) and 3 ▸(*c*).

SESANS with linear shifters, shown schematically in Fig. 1 ▸(*b*), satisfies the spin echo condition for the unscattered neutron beam, and the labelling of the scattering angle or momentum transfer is along the direction of the magnetic field integral gradient. Consequently, the combination of linear shifters and longitudinal precession fields reproduces the conventional linear SESANS technique and may be considered as an add-on option for NSE spectrometers.

The SESANS method discussed here uses radial shifters, and the resulting magnetic field integral is therefore rotationally symmetric about the optical axis. In the following we evaluate the performance and capabilities of radial SESANS assuming that the magnetic field configuration of the host NSE spectrometer is ideal, *i.e.* the magnetic field integral is the same for all possible neutron trajectories before and after the sample. This is technically achievable using correction elements, in particular Fresnel coils (Monkenbusch, 1990 ▸). Thus, the host NSE spectrometer ideally satisfies the spin echo condition φ = 0 for both the non-scattered and the elastically scattered (ω = 0) neutron beams considered here. For this reason, in the following we will make an abstraction of the host instrument magnetic field configuration and will consider solely the effect of additional coils, such as linear or radial shifters, on φ for both the non-scattered and the elastically scattered neutron beams.

Ideally, Fresnel coils can be considered as consisting of concentric current loops, as shown schematically in Fig. 3 ▸(*a*), generating a magnetic field integral that counterbalances the intrinsic *r* ^2^ dependence of the magnetic field integral produced by solenoids (Mezei, 1980*a* ▸; Monkenbusch, 1990 ▸). In this way, NSE spectrometers can reach long Fourier times while keeping large beam cross sections and thus without sacrificing their data acquisition rate.

Shifters are similar to Fresnel coils but they generate a magnetic field integral proportional to a characteristic distance, *e.g.* along *x* as in Fig. 3 ▸(*b*) or along the radius as in Fig. 3 ▸(*c*). Linear shifters as in Fig. 3 ▸(*b*) were introduced in the early days of NSE spectroscopy to produce linear gradients in the magnetic field integral that can map the *Q*-dependent energy (dispersion) of collective excitations (Mezei, 1980*b* ▸).

The focus here is on radial shifters, the magnetic field integral of which varies linearly with the radius. In this case the resulting precession phase for neutrons crossing a radial shifter at a radius *r* is given by 

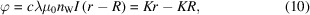

with μ_0_ the magnetic permeability, *n*_W_ the winding density, *i.e.* the number of wires per unit length, *R* the outer radius of the radial shifter (*r* < *R*) and *I* the current in the coil. From now on we will ignore the term *KR* since it will in all configurations be compensated by the same term in another radial shifter with opposite sign. All tuning parameters are summarized in the constant *K*. For example, for a monochromatic neutron beam with λ = 10 Å and a shifter with *n*_W_ = 2 mm^−1^ and *I* = 10 A, *K* = 11651 m^−1^.

In the following we assume that the neutron beam is perfectly monochromated. However, in the absence of scattering the spin echo condition is wavelength independent, and for this reason a wavelength distribution does not affect the performance of the setup but leads only to a distribution of spin echo lengths (Rekveldt *et al.*, 2005 ▸). In SEMSANS this distribution would decrease the amplitude for the higher-order modulations.

In our calculations the beam divergence is defined by the diaphragms. For an improved data acquisition rate one could envisage focusing the beam at the sample position. This would be possible by adequate design of the neutron delivery system, *e.g.* using a focusing mirror, as foreseen on IN15 (Schleger *et al.*, 1999 ▸).

The performance of SESANS as given by equation (10) is independent of the main magnetic field from the host NSE instrument, which in principle could stay constant during the SESANS measurements. In this way it would be easy to disentangle the SESANS signal from any inelastic scattering. The latter would reduce the amplitude of the modulation and could be determined by NSE measurements with zero current in the shifter coils.

**Figure 4:**
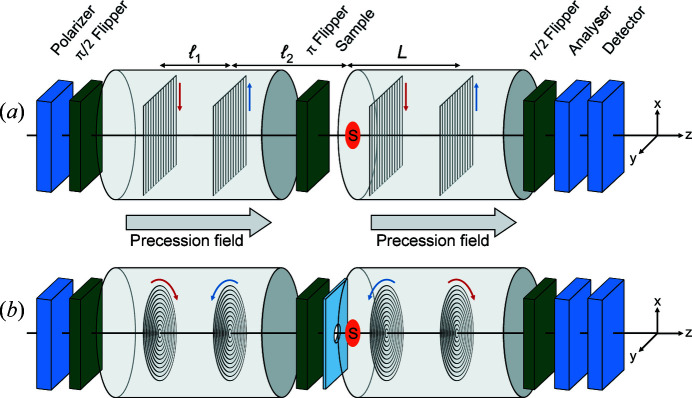
Schematic drawings of the SESANS instruments discussed here, with (*a*) linear and (*b*) radial shifters. The shifters are positioned in the main precession fields at a distance ℓ_1_ from each other. The direction of the current in the shifters is indicated by arrows. The π flipper in front of the sample represents the symmetry point of the setup.

Fig. 4 ▸ provides a schematic illustration of the linear and radial SESANS setups discussed here, which are conceptually equivalent to those of Figs. 1 ▸(*b*) and 1 ▸(*c*). The precession field areas are positioned on each spectrometer arm symmetrically around the π flipper, which is in the centre of the setup and next to the pinhole at the sample position. The precession fields are created by coils and thus point towards the optical axis *z*. The labelling of the neutron beam trajectories is performed by two pairs of shifters, one at each arm of the setup, and the shifters of each pair are at a distance ℓ_1_ apart from each other. Furthermore, the inner shifters, which are positioned next to the pinhole at the sample position, are at a distance ℓ_2_ from it. Thus, the total distance between the outer shifters and the pinhole is *L* = ℓ_1_ + ℓ_2_. The arrows indicate the direction of the electric currents in the shifters. Ideally, both the π flipper and the pinhole should be positioned at the geometric centre of the setup. However, as the pinhole has finite dimensions its exact positioning will not be critical for the final performance of the setup.

### 2.1. Definition of neutron beam trajectories

**Figure 5:**
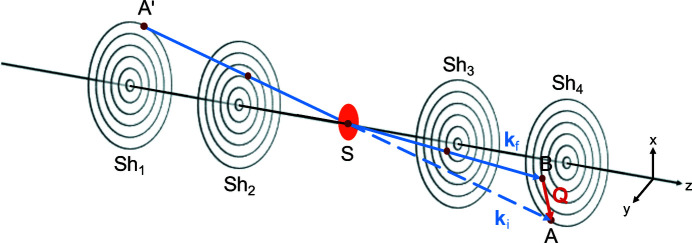
A schematic drawing of a possible neutron trajectory through the four shifters of the setup with scattering at the sample. The wavevectors **k**_i_ and **k**_f_ are defined for the direct and scattered beam, respectively. The momentum transfer **Q** is given as their difference.

In the following we focus on the performance of radial SESANS and we start by defining the neutron beam trajectory coordinates, expressing them in terms of the beam’s crossing points with the four shifters (Sh) and the pinhole (S), as illustrated in Fig. 5 ▸. For this purpose we will use cylindrical coordinates, which are concordant with the symmetry of the setup. The coordinate system is defined such that the *z* axis coincides with the optical axis of the instrument and the pinhole is located at the origin. Without any loss of generality, the crossing points are given by 

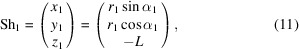




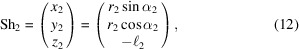




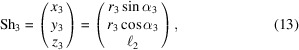




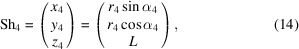




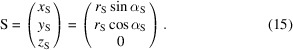

**Figure 6:**
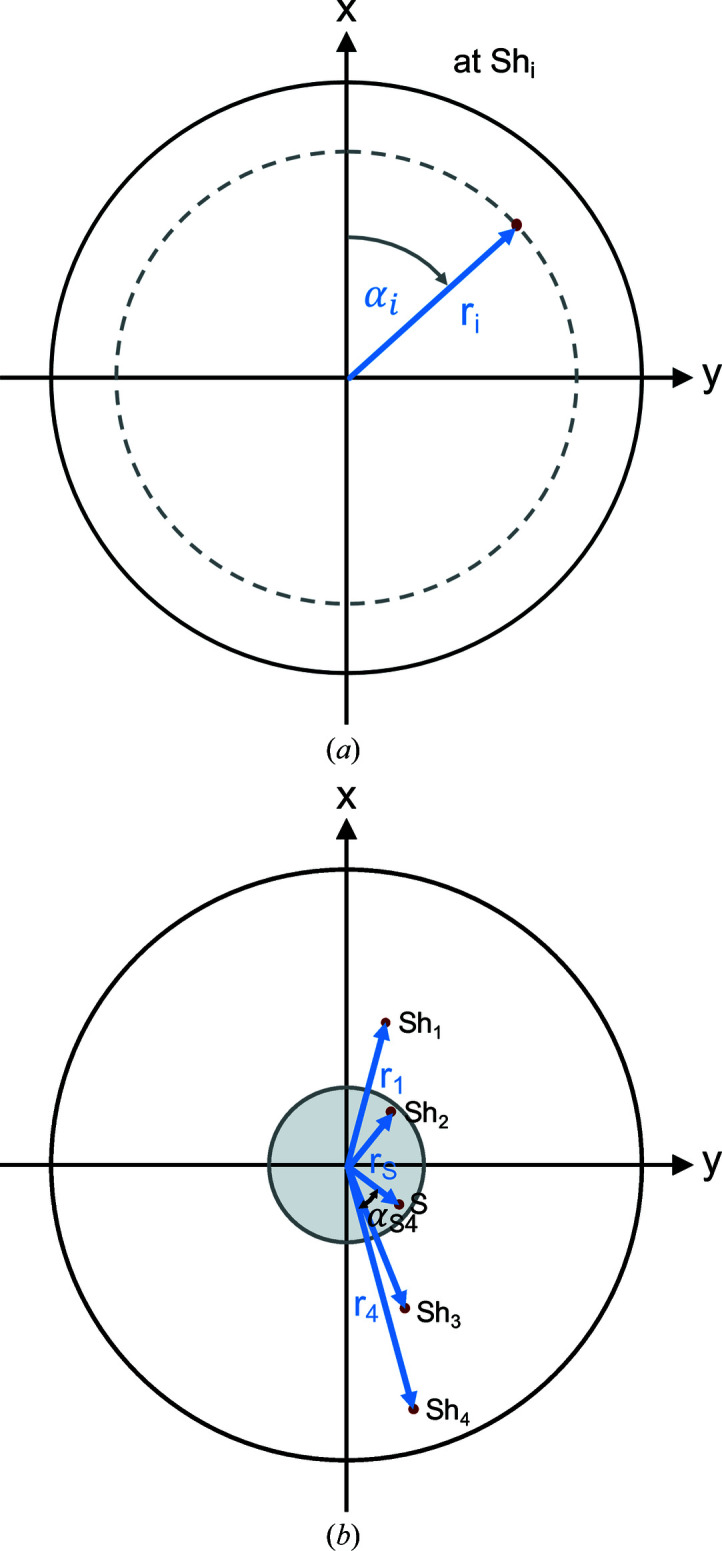
(*a*) The definition of the crossing point of a neutron with shifter Sh_*i*_ in polar coordinates (*r*_*i*_, α_*i*_). (*b*) A projection of a direct beam trajectory on the shifter plane to illustrate the relationship between the radial coordinates from equations (11)–(15) [Disp-formula fd13] [Disp-formula fd14] [Disp-formula fd15]. The coordinate *r*_S_ at the sample is restricted to the pinhole area with radius *p* which is indicated in grey.

Here *r*_*i*_ is the radius of the crossing point at shifter Sh_*i*_ or at the pinhole, and α_*i*_ are the corresponding angles in the *xy* plane, as shown in Fig. 6 ▸.

In the absence of scattering, two points are sufficient to define the trajectory through the whole setup. Thus, *r*_1_, *r*_2_ and *r*_3_ can be expressed as a function of the coordinates at the sample S and the fourth shifter Sh_4_: 

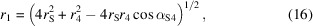




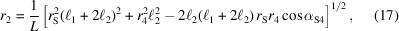




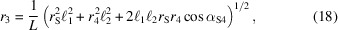

with α_S4_ = α_S_ − α_4_ and *L* = ℓ_1_ + ℓ_2_. In the case where 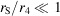 , *i.e.* when the radius of the beam at the sample position is small compared with that at the shifters, we can simplify the above expressions using the Taylor expansion: 

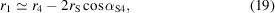




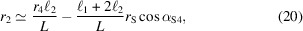




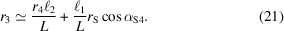




### 2.2. Neutron beam trajectory and Larmor precession

In general, the total precession angle Δφ is equal to the difference between the precession angle before and after the π flipper. In this SESANS setup, Δφ depends only on the contributions of the four shifters (φ_*i*_, *i* = 1,…, 4) that encode the neutron trajectory, leading to

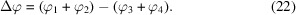

Here the relative orientation of the electric currents in the shifters, which we will designate in the following by their signs (+ and −), affects the sign of the corresponding φ_*i*_. In the absence of scattering, the linear SESANS (+−, +−) configuration shown in Fig. 4 ▸(*a*) satisfies the spin echo condition for all beam sizes and divergences.

The case of radial SESANS, however, is more restrictive as the beam dimensions at the sample position have a significant impact on the performance of the setup. This is most prominent for the (++, ++) configuration [equivalent to Figs. 1(*b*) and 1(*c*) in the report by Zhao (2001 ▸)], the net precession angle of which can be calculated using the approximations of equations (19)–(21) [Disp-formula fd21]: 

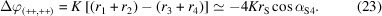

**Figure 7:**
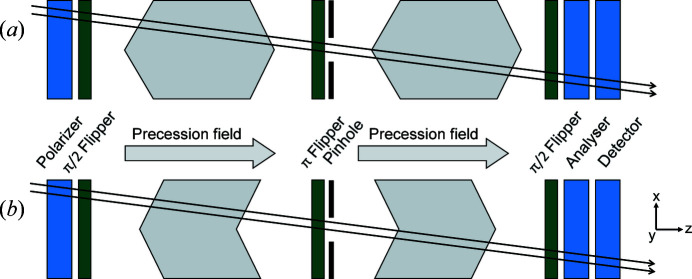
Schematic drawings of the precession path through the two different configurations for two different neutron trajectories. The *r*_S_ direction is taken along the *y* direction (perpendicular to the paper). We consider only the trajectory for α_S4_ = 0 or π, which means that all *r*_*i*_ are in the *x* direction. (*a*) In the (++, ++) configuration the path lengths through the first and second precession regions are only equal for trajectories going exactly through the centre of the setup, as illustrated for the lower trajectory. In the higher trajectory the path length through the first precession region becomes shorter, while it becomes longer for the second region, as reflected in equation (23). (*b*) In the (+−, −+) configuration the exact value of *r*_S_ has no effect on the echo. For a certain angle of the trajectory it does not matter if the neutron path is higher or lower, as long as the *x* components of the radii in the shifters change sign from the first precession region with respect to the second precession region, as reflected in equation (24).

This result can be understood from the geometry of the precession regions. Considering the case of α_S4_ = π/2 or 3π/2 in Fig. 6 ▸ will result in a symmetric situation for the radii of the trajectory in the first arm as in the second arm. In that case we expect an echo, which is illustrated mathematically by the fact that in those cases 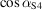 = 0. On the other hand, when α_S4_ = 0 or π, then the asymmetry will be maximal. Only for *r*_S_ = 0 will there be an echo, as sketched in Fig. 7 ▸(*a*). This implies that this configuration satisfies the spin echo condition only for infinitesimally small pinholes, *i.e.* when the neutron trajectory passes exactly though *r*_S_ = 0, and is very sensitive to deviations from it. Any realistic pinhole would therefore lead to significant deviations from the spin echo condition and a loss of the spin echo modulation. This is a severe drawback and for this reason, in the following, we will discard this configuration and focus on the performance of the (+−, −+) setup. In that case, and in the presence of a pinhole with radius *p* at the sample position, so that 0 ≤ *r*_S_ ≤ *p*, following equations (10) and (22) the net precession angle can be written assuming the approximations of equations (19)–(21) [Disp-formula fd21]: 

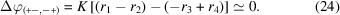

This result can also be understood from the geometry of the setup as sketched in Fig. 7 ▸(*b*) by considering only the most asymmetric case when α_S4_ = 0 or π. For trajectories where the signs of the *x* components of the radii of the first two shifters are both opposite to those of the last two shifters this will always result in an echo situation. The path length through either of the two precession regions is only dependent on the angle of the trajectory and not on its position. This implies that, in the absence of scattering, the spin echo condition is satisfied in this configuration for any small pinhole of finite size.

The resulting NSE polarization in the most general case, beyond the small sample pinhole approximation, can be calculated by taking all possible neutron trajectories into account and is given by

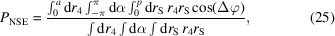

where *p* and *a* are the maximum pinhole and shifter radii, respectively, α = α_S4_, and the net precession angle Δφ is given by equations (24) and (16)–(18) [Disp-formula fd18].

**Figure 8:**
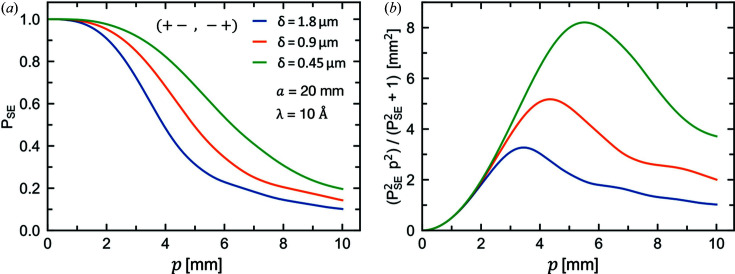
(*a*) Spin echo polarization and (*b*) FOM in the absence of scattering as a function of the pinhole radius *p* for *a* = 20 mm, λ = 10 Å and different spin echo lengths δ. The *P*_NSE_ and the FOM are derived from equations (25) and (26), respectively.

Fig. 8 ▸(*a*) depicts the effect of the pinhole radius on *P*_NSE_, as obtained by numerical integration for *a* = 20 mm, λ = 10 Å and different spin echo lengths δ. A radius of 20 mm is a reasonable assumption; smaller values would of course be possible but would also lead to a dramatic decrease in the data acquisition rate.

Only an infinitesimally small pinhole (*p* = 0) leads to *P*_NSE_ = 1, thus only this case satisfies the spin echo condition perfectly. By increasing the pinhole size, an increasing number of neutron trajectories do not satisfy the spin echo condition, leading to a reduction in *P*_NSE_. This reduction is substantial, but not obstructive, as a pinhole with a radius of 5 mm (thus *a*/4) leads to *P*_NSE_ ≃ 0.6, which, depending on the spin echo length of the measurement, would be sufficient for performing SESANS measurements.

For a more quantitative evaluation we calculated the figure of merit (FOM) of the setup, derived from the relative error of *P*_NSE_: 

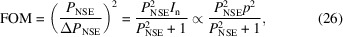

where the error Δ*P*_NSE_ is determined from the derivative of *P*_NSE_ and *I*_n_ is the total intensity of the detected neutron beam, proportional to the area of the pinhole, *I*_n_ ∝ *p* ^2^. As shown in Fig. 8 ▸(*b*), if the FOM is plotted against the pinhole size, it goes through maxima which mark the optimal operating conditions with the best compromise between resolution and neutron beam intensity.

### 2.3. Effect of scattering: relation between precession and momentum transfer

In the case of scattering, the trajectories before and after the sample are no longer correlated with each other. Here we start by assuming an infinitesimally small pinhole at the sample position such that *r*_S_ = 0. In this case there is a clear relation between the radii of the points where the neutron beam impinges on the shifters: 

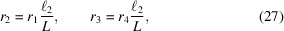

where in the absence of scattering *r*_1_ = *r*_4_ and *r*_2_ = *r*_3_. Using equation (27), the net precession angle defined in equation (24) becomes

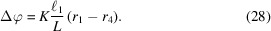




Fig. 5 ▸ shows the trajectory of a possible scattering process and defines the crossing points at the shifters. The direct neutron beam travels from *A*′ at the first shifter to point *A* at the fourth shifter, with 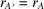 because of the pinhole. Scattered neutrons are deflected to point *B*.

The momentum transfer is defined as **Q** = **k**_i_ − **k**_f_, where **k**_i_ and **k**_f_ are the wavevectors of the incident and scattered neutrons, respectively. Only elastic scattering is considered here and therefore |**k**_i_| = |**k**_f_| = *k*_0_ = 2π/λ. The vector **k**_i_ points along the trajectory of the incident or direct beam, along the vector **SA** from the crossing point with the sample S to the point *A* (equal to the vector between *A*′ at the first shifter and S), while the direction of **k**_f_ is given by the vector **SB** from S to *B*: 

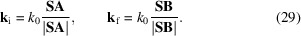




Using the coordinate notation from above for the crossing points *A* and *B*, and the condition for a pinhole (*p* = 0), the wavevectors can be written as 

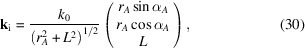




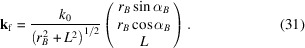

Consequently, the total momentum transfer vector becomes 

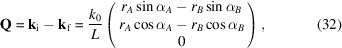

where the square roots are approximated assuming 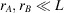 , such that (*r* ^2^ + *L* ^2^)^1/2^ ≃ *L*, and the modulus of the momentum transfer is given by

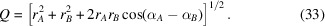




Both the equation for **Q** and Fig. 5 ▸ show that the momentum transfer takes place in the *xy* plane. This is the detector plane, and looking at the scattering process in this plane involves the projected wavevectors derived from equations (30) and (31): 

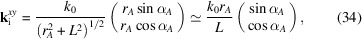




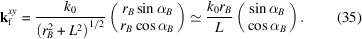




The moduli of these vectors depend on the radial coordinates *r*_*A*_ and *r*_*B*_: 

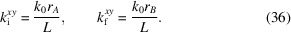




**Figure 9:**
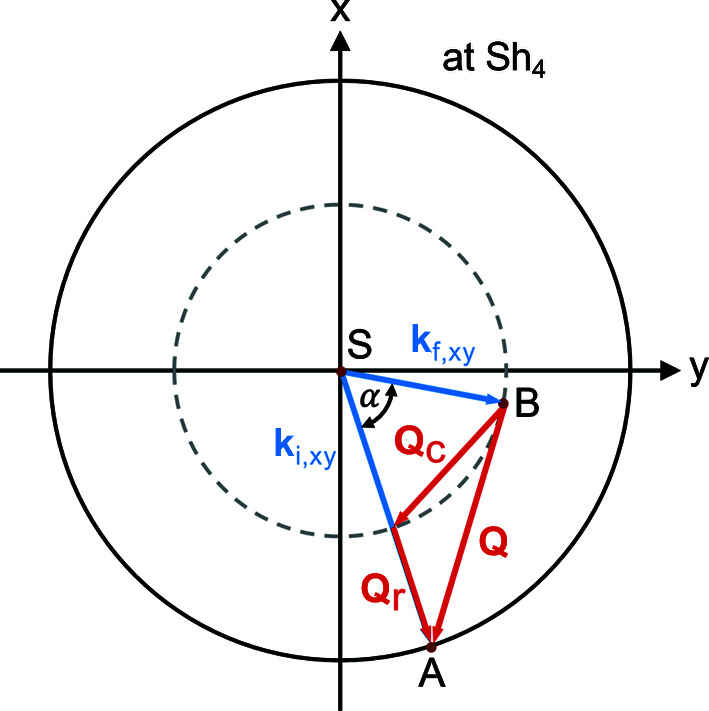
The scattering event shown in Fig. 5 ▸ as seen from the sample, projected onto the *xy* plane. The total momentum transfer **Q** is decomposed into a radial component **Q**_r_ and a complementary component **Q**_C_. Only the magnitude of **Q**_r_ is included in the measured precession angle. The angle α denotes the difference between the polar angle at the first and last shifter, α = α_1_ − α_4_.

The total momentum transfer **Q** is now decomposed into a radial **Q**_r_ and a complementary **Q**_C_ component, as shown in Fig. 9 ▸. **Q**_r_ points in a radial direction parallel to 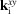 and its modulus is determined by the lengths of the wavevector projections given in equation (36). The difference between 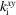 and 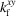 labels the momentum transfer in the radial direction: 

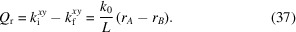

In contrast, the complementary component cannot be expressed clearly in terms of the wavevectors without knowing the polar coordinates α_*A*_ and α_*B*_.

By combining the derived expressions for the precession angle Δφ [equation (28)] and *Q*_r_ [equation (37)] while considering that *r*_*A*_ = *r*_1_ and *r*_*B*_ = *r*_4_, we find that the net precession angle is proportional only to the radial momentum transfer. This is an important result, indicating that only one component of the momentum transfer vector is included in the measured signal. Since there is no unique relationship between **Q**_r_ and **Q**_C_, it is not possible to calculate the magnitude or direction of **Q** from the vector equation **Q**_r_ + **Q**_C_ = **Q** without knowledge of **Q**_C_. In other words, radial SESANS probes only the momentum transfer along the radial direction without the possibility of obtaining the total momentum transfer, 

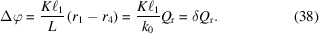

This equation has been derived in the limit of an infinitely small sample pinhole. A satisfactorily high *P*_NSE_ can be obtained for a sample pinhole with a diameter *p* = *a*/4. Therefore, equation (38) will be less strict for scattering events, especially those where the incoming and outgoing vectors are close to the optical axis. However, these events occur less frequently than those where they are closer to the edge of the diaphragms, since there is more area. This small deviation from equation (38) gives rise to a spread in precession for a certain *Q*_r_, leading to a resolution effect for the SESANS measurement as a function of spin echo length.

As in linear SESANS, the proportionality factor δ in equation (38) is the spin echo length, 

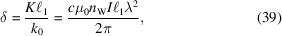

which can be varied by changing the current *I* in the shifter coils while keeping all other parameters, including the precession magnetic field, unchanged.

The NSE polarization and FOM in Fig. 8 ▸ were calculated for different δ values that can be achieved with the described setup. For *I* varying between 2 and 10 A in shifter coils with a winding density of *n*_W_ = 2 mm^−1^, ℓ_1_ = 1 m and a neutron wavelength of λ = 10 Å, δ ranges from 0.37 to 1.85 µm.

**Figure 10:**
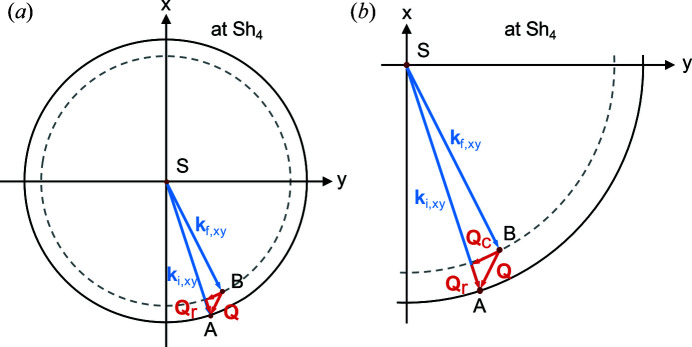
(*a*) A scattering event with a small momentum transfer projected onto the *xy* plane and (*b*) an enlargement of the lower right quadrant. In this case, **Q**_r_ and **Q**_C_ are approximately perpendicular to each other, which corresponds to the situation in linear SESANS.

In general, the defined momentum transfer components **Q**_r_ and **Q**_C_ are not perpendicular to each other. For scattering with a small total momentum transfer, however, this can be assumed since *Q*_r_ is small and a linear approximation, as shown in Fig. 10 ▸, is justified. If so, 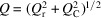 holds for the measured component *Q*_r_ and the complementary component *Q*_C_. This situation resembles the momentum encoding in linear SESANS, where the total momentum transfer is always decomposed into perpendicular components. In other words, radial SESANS is similar to the conventional linear method, as it does not allow for accessing more than just a component of the momentum transfer vector.

A consequence of the fact that Δφ depends only on **Q**_r_ is that radial SESANS probes only correlations along the radial direction. Similar to linear SESANS, this leads to a projected correlation function, defined as the Hankel transform of the sample’s scattering function. For radially symmetric functions the Hankel transform can be derived from a two-dimensional Fourier transform (Bouwman *et al.*, 2000 ▸; Andersson *et al.*, 2008 ▸; Uca *et al.*, 2003 ▸), 

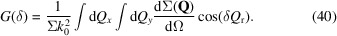

The integral is over all *Q* values reaching the detector. In the given case, the integral translates to an integral over the radii of the first and last shifter and over the difference in polar angles α (*cf*. Fig. 9 ▸). The normalized correlation function becomes 

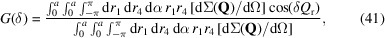

where *a* is the shifter radius determining the maximum detectable *Q* value and thus the minimum size measurable.

### 2.4. Scattering from spherical particles

In the following we evaluate the performance of the radial SESANS setup and compare it with linear SESANS. For this purpose we consider the simple case of isolated spherical particles of radius *R*, whose scattering function is given by (Guinier & Fournet, 1955 ▸)

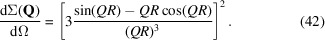

**Figure 11:**
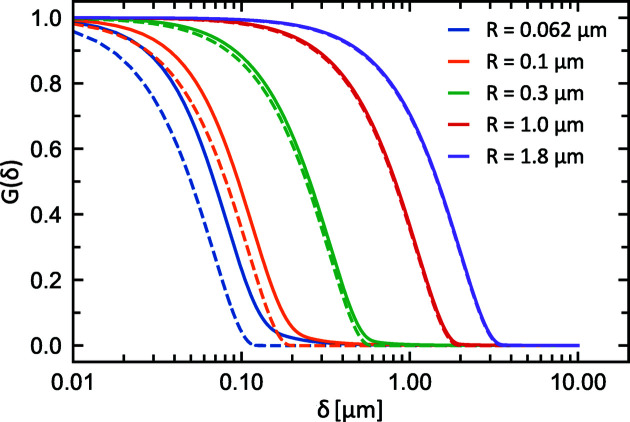
Comparisons of the correlation function *G* of radial and linear SESANS, calculated for dilute spherical particles of different radii *R*. The solid lines represent *G* for radial SESANS as a function of the spin echo length δ, while the dashed lines are the corresponding *G*(δ) for linear SESANS. The minimum measurable length scale in radial SESANS is determined by the maximum *Q* value detected, which depends on the scattering angles and the size of the detector.

The results for different particle radii are drawn in Fig. 11 ▸ for both radial and linear SESANS. For the latter, *G*(δ) has been calculated analytically (Krouglov, de Schepper *et al.*, 2003 ▸) and thus corresponds to an ideal setup where instrument parameters, such as angular acceptance, are not taken into account.

Comparison of the two data sets shows that *G*(δ) of radial SESANS also goes to zero at the δ values corresponding to the maximal distance of the scattering particle, which in the case of spheres corresponds to their diameter. On the other hand, for small *R*, and therefore large values of *Q*, the radial SESANS results deviate from those of the analytical calculation. This is due to the angular acceptance of the setup and thus the shifter size, which determines the maximal detectable *Q* value in radial SESANS. The calculation of equation (41) was performed with a shifter radius of *a* = 20 mm, which gives a minimum measurable particle radius of less than *R* = 50 nm for the given setup (ℓ_1_ = ℓ_2_ = 1 m, λ = 10 Å). Below this radius the SESANS correlation function deviates from the analytical result, and this holds not only for radial but also for linear SESANS (Rekveldt, 1996 ▸).

## 3. Radial SEMSANS

**Figure 12:**
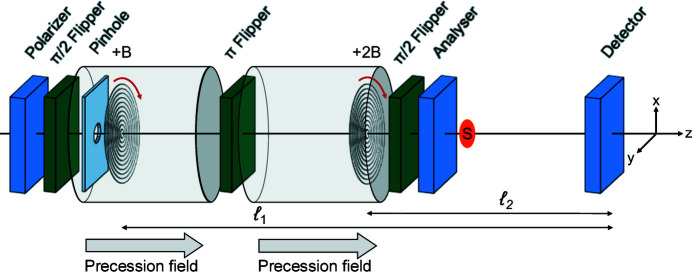
A schematic drawing of the radial SEMSANS instrument. The second shifter generates a magnetic gradient twice as strong as the first one, resulting in a modulation of the beam intensity at the detector in a radial direction.

The rotationally symmetric magnetic field geometry, consisting of radial shifters and longitudinal precession fields, can also be implemented for SEMSANS. In this technique, all spin manipulations take place in two precession regions before the sample (see Fig. 12 ▸).

Existing SEMSANS instruments are built with transverse precession fields that have inclined front and end faces, as for linear SESANS. In this case, the total Larmor precession angle accumulated by the neutrons depends linearly on the height of their trajectory at the detector but is independent of their divergence angle. When the focus condition *B*_1_ℓ_1_ = *B*_2_ℓ_2_ is satisfied (Bouwman *et al.*, 2009 ▸, 2011 ▸), where *B*_1_ and *B*_2_ are the precession magnetic fields and ℓ_1_ and ℓ_2_ are the distances of the respective precession areas from the detector, this leads to a spatial modulation of the neutron beam intensity at the detector. Any scattering by a sample decreases the amplitude of that modulation, also called visibility (Bouwman *et al.*, 2011 ▸; Li *et al.*, 2016 ▸).

The SEMSANS normalized visibility is analogous to the normalized NSE polarization in SESANS, and therefore both methods probe the same projected correlation function (Bouwman *et al.*, 2011 ▸; Strobl *et al.*, 2015 ▸). The labelled direction in the precession angle φ, and thus the orientation of the modulation, are determined by the direction of the magnetic fields. While transverse fields create a modulation along the detector height (transverse direction), the modulation in radial SEMSANS would be along the radial coordinate, starting at the beam centre.

### 3.1. Precession angle and momentum transfer

Fig. 12 ▸ shows the setup of a radial SEMSANS instrument. Two shifters encode the radial coordinate of the neutron trajectory in φ. The magnetic fields and positions are set to *B*_2_ = 2*B*_1_ and ℓ_1_ = 2ℓ_2_ to satisfy the focus condition. As before, a neutron trajectory can be defined by its crossing points at the shifters Sh_*i*_, the sample S and the detector D using cylindrical coordinates: 

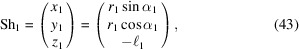




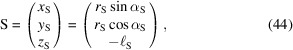




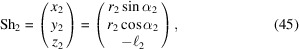




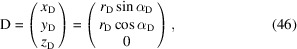




Using equation (10), the total precession angle Δφ, defined as the difference between the precession accumulated before and after the π flipper, becomes 

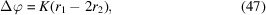

in which *K* is defined as in equation (10) for the first shifter. In SEMSANS it is crucial to have a direct proportionality between Δφ and the radial position of a neutron at the detector *r*_D_. In the absence of scattering, the radial coordinate of the crossing point at the second shifter *r*_2_ can be first expressed as a function of *r*_1_ and *r*_D_. With ℓ_1_ = 2ℓ_2_ and α_1D_ = α_1_ − α_D_ this gives 

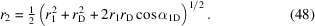

As a second step we introduce a small pinhole in front of the first shifter, so that, 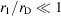 and we can use the Taylor expansion,

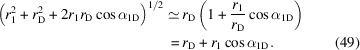

This simplifies the expression for the total precession angle, which in the absence of scattering becomes

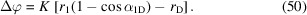

This indeed provides a linear relation between Δφ and *r*_D_, which shows that this setup creates a radial modulation. However, this result is not valid close to the detector centre (*r*_D_ → 0) where our approximation using the Taylor expansion breaks down.

In the presence of scattering, we can express Δφ as a function of the radial momentum transfer *Q*_r_ and a spin echo length in a way very similar to radial SESANS. For this derivation, we introduce an infinitesimally small pinhole in front of the first shifter so that *r*_1_ → 0. The radius *r*_2_ can then be derived from the geometry of the setup: 

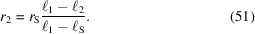

The precession angle from equation (47) becomes 

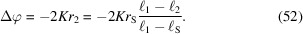

Considering the wavevectors for the incident and scattered neutron beams, we derive the moduli of their projections onto the *xy* plane as

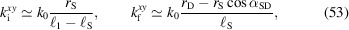

where α_SD_ = α_S_ − α_D_, and *r*_S_ and *r*_D_ are small compared with ℓ_1_ or ℓ_S_.

The radial component of the momentum transfer vector thus becomes 

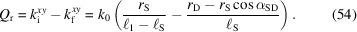

Using equations (52) and equation (54) we obtain 

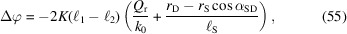

where the factor in front of *Q*_r_ denotes a spin echo length δ, with 

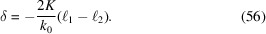

The spin echo length depends on the distance between the shifters, which is as expected and is also the case for radial SESANS.

The variation in the visibility of the modulation in the beam intensity at the detector can be calculated in the most general case, beyond the small pinhole approximation. For a pinhole of radius *R* in front of the first shifter, so that 0 ≤ *r*_1_ ≤ *R*, we obtain

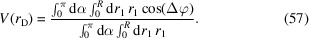

**Figure 13:**
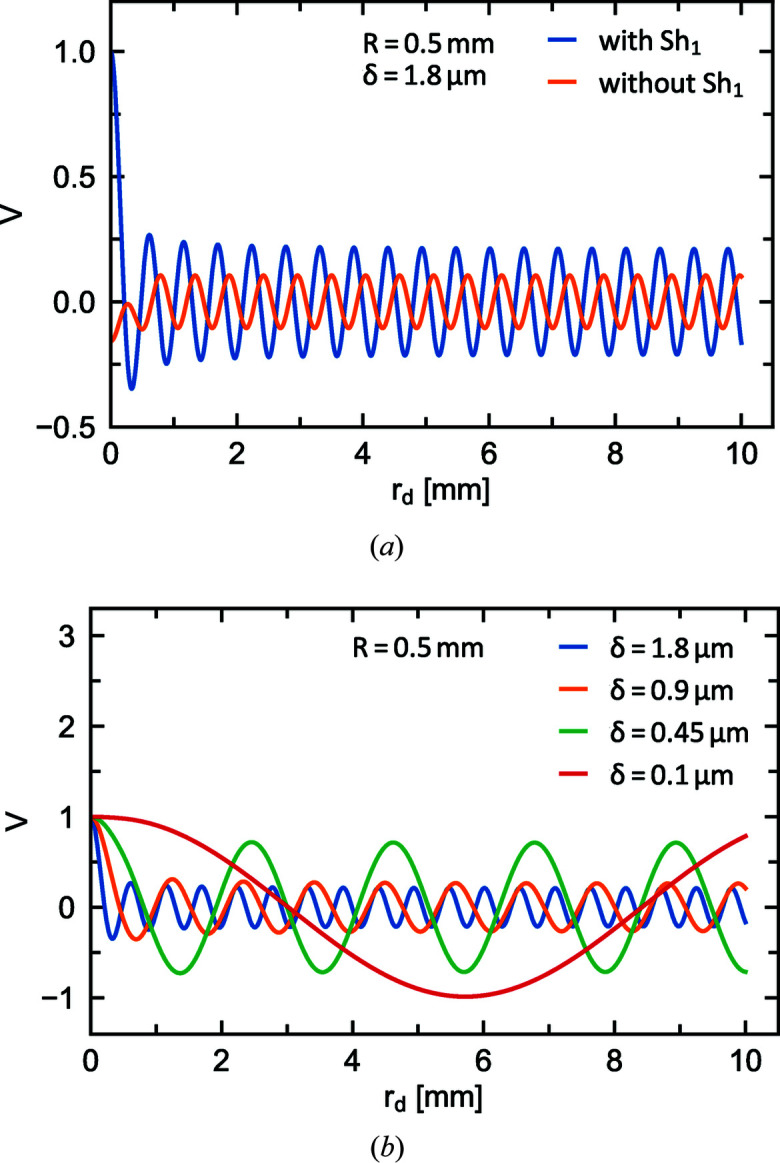
The normalized visibility *V* of the intensity modulation as a function of the radial distance from the detector centre *r*_D_. *V* was calculated with equation (57) for a pinhole radius of *R* = 0.5 mm. (*a*) Modulation visibility with and without the first shifter. If the pinhole is smaller than the first loop of the shifter coil, the magnetic field of Sh_1_ does not affect the neutrons, as if no shifter were present. (*b*) *V* for different values of spin echo length.

In the following, Δφ was determined using equations (47) and (48) in equation (57). The integration was performed numerically for different spin echo lengths between 0.1 and 1.8 µm. The results obtained in the absence of scattering, for a pinhole with *R* = 0.5 mm and as a function of *r*_D_, are illustrated in Fig. 13 ▸. This shows that it is also possible to perform SEMSANS measurements with radial shifters, even though the visibility is lower than for linear SEMSANS.

As in the previous section for SESANS, we consider here shifters with a winding density of *n*_W_ = 2 mm^−1^. The generated magnetic field gradient is therefore not continuous, as assumed in the mathematical model, but a stepped function of the windings. This approach thus breaks down when the radius of the pinhole becomes comparable to or smaller than the radius of the innermost shifter coil loop. In this case the effect of the first shifter drops out, leading to another expression for the total precession angle: Δφ = −2*K* *r*_2_. The effect on the modulation can be seen in the recalculated visibility shown in Fig. 13 ▸(*a*). This effect will further reduce the visibility for radial SEMSANS.

## 4. Concluding remarks

Since the early days of NSE it has been acknowledged that shifter coils combined with longitudinal precession fields, as found in NSE spectrometers, may be used to encode the neutron beam trajectories for SESANS-like applications (Pynn, 1978 ▸, 1980 ▸). In this article we have discussed how linear shifter coils can lead to a configuration that is conceptually equivalent to the SESANS setup, which has been developed in Delft (Rekveldt, 1996 ▸; Rekveldt *et al.*, 2003 ▸; Andersson *et al.*, 2008 ▸) and uses transverse (vertical) magnetic fields with inclined boundaries. This configuration is only sensitive to the projection of the momentum transfer vector along the (vertical) direction, which is defined by the inclination of the magnetic field boundaries.

Here we have considered radial, instead of linear, magnetic field integral gradients and have evaluated the performance of a SESANS setup resulting from the combination of radial shifter coils with longitudinal precession fields, in concordance with the cylindrical symmetry of the setup. Our results show that this configuration is also sensitive to the projection of the momentum transfer vector along one direction, in this case the radial one. Thus, in this case SESANS also yields a projected correlation function. We argue that this should be the case for all SESANS realizations, independent of their specific magnetic field geometry. In fact, as the magnetic field integral varies in a continuous fashion across the area of the neutron beam it is always possible to identify continuous ‘equipotential’ lines along which the magnetic field (integral) gradient vanishes [*e.g.* along *x* in Fig. 4 ▸(*a*) or along all circles centred at *y* = 0 and *z* = 0 in Fig. 4 ▸(*b*)]. Consequently, the SESANS signal would be insensitive to the correlation function along these lines and would yield the projected correlation function along those direction(s) along which the magnetic field (integral) gradient is maximum: *y* in Fig. 4 ▸(*a*) or the radial direction in Fig. 4 ▸(*b*). This is an important difference between SESANS and NSE spectroscopy and explains why the former yields a projected correlation function, whereas the latter, which probes a scalar quantity (the energy transfer at the sample), delivers the intermediate scattering function directly.

The performance of linear SESANS does not depend on neutron beam characteristics such as beam dimensions and divergence. In contrast, our results show that radial SESANS performs best for 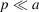 , *i.e.* when the radius of the neutron beam at the sample is small in comparison with that at the outer shifter coils. This requirement ensures that the spin echo condition is satisfied for a majority of trajectories but also leads to substantial intensity losses. Nonetheless, the results of Fig. 8 ▸ show that for a realistic configuration, in the absence of scattering, it is possible to increase the beam size at the sample, *e.g.* *p*/*a* ≃ 1/4, while keeping a reasonable spin echo polarization. In addition, scattering from a model sample consisting of simple spherical particles provides results consistent with what would be expected for linear SESANS. The simple setup considered here should reach spin echo lengths ranging from about 100 nm to a few micrometres. Higher resolutions could be obtained by using stacked shifter coils, as is commonly the case with Fresnel coils in NSE spectrometers.

Longitudinal magnetic fields combined with radial shifter coils could also be used for radial SEMSANS, although with a substantially reduced performance with respect to its linear SEMSANS counterpart. Radial SEMSANS requires the smallest possible beam aperture at the point (first shifter) where the Larmor labelling begins. This of course dramatically reduces the intensity of the neutron beam and at the same time makes the first shifter redundant when the dimensions of the aperture become comparable to the distance between the wires of this shifter coil.

To conclude, linear and radial SE(M)SANS are conceptually similar to each other but the linear realization clearly outperforms the radial one. In addition, both setups can be realized by combining shifter coils (radial or linear) with longitudinal magnetic fields, as found at NSE spectrometers, and could be implemented as add-ons to existing instruments. The spin echo length that can be reached would be similar to those obtainable on present SESANS instruments (some 10–20 µm). The advantage of such a realization would lie in the combination of the long wavelengths accessible to existing NSE instruments and their high neutron flux. In this way it would be possible to study weakly scattering systems and perform kinetic measurements on the timescale of minutes instead of hours, which is presently the limit.

Compared with existing SESANS instruments, such a realization would allow for using custom-made in-beam coils, with current densities adapted to specific requirements that could enable new types of experiments with unexplored capabilities.
